# 670 nm light mitigates oxygen-induced degeneration in C57BL/6J mouse retina

**DOI:** 10.1186/1471-2202-14-125

**Published:** 2013-10-17

**Authors:** Rizalyn Albarracin, Riccardo Natoli, Matthew Rutar, Krisztina Valter, Jan Provis

**Affiliations:** 1 ARC Centre of Excellence in Vision Science and John Curtin School of Medical Research, 131 Garran Road, Canberra, ACT 0200, Australia; 2 ANU Medical School, The Australian National University, Canberra, ACT 0200, Australia

**Keywords:** 670 nm light irradiation, Near infrared, Photobiomodulation, Hyperoxia, Retinal degeneration, Neuroprotection, Cytochrome oxidase, Oxidative stress, Retinal inflammation, Oxygen toxicity

## Abstract

**Background:**

Irradiation with light wavelengths from the far red (FR) to the near infrared (NIR) spectrum (600 nm -1000 nm) has been shown to have beneficial effects in several disease models. In this study, we aim to examine whether 670 nm red light pretreatment can provide protection against hyperoxia-induced damage in the C57BL/6J mouse retina. Adult mice (90–110 days) were pretreated with 9 J/cm^2^ of 670 nm light once daily for 5 consecutive days prior to being placed in hyperoxic environment (75% oxygen). Control groups were exposed to hyperoxia, but received no 670 nm light pretreatment. Retinas were collected after 0, 3, 7, 10 or 14 days of hyperoxia exposure (n = 12/group) and prepared either for histological analysis, or RNA extraction and quantitative polymerase chain reaction (qPCR). Photoreceptor damage and loss were quantified by counting photoreceptors undergoing cell death and measuring photoreceptor layer thickness. Localization of acrolein, and cytochrome *c* oxidase subunit Va (Cox Va) were identified through immunohistochemistry. Expression of heme oxygenase-1 (*Hmox-1*), complement component 3 (*C3*) and fibroblast growth factor 2 (*Fgf-2*) genes were quantified using qPCR.

**Results:**

The hyperoxia-induced photoreceptor loss was accompanied by reduction of metabolic marker, Cox Va, and increased expression of oxidative stress indicator, acrolein and *Hmox-1*. Pretreatment with 670 nm red light reduced expression of markers of oxidative stress and *C3*, and slowed, but did not prevent, photoreceptor loss over the time course of hyperoxia exposure.

**Conclusion:**

The damaging effects of hyperoxia on photoreceptors were ameliorated following pretreatment with 670 nm light in hyperoxic mouse retinas. These results suggest that pretreatment with 670 nm light may provide stability to photoreceptors in conditions of oxidative stress.

## Background

Irradiation with light wavelengths from the far red (FR) to the near infrared (NIR) spectrum (600 nm -1000 nm) has been shown to have beneficial effects in mammalian tissues [1-5]. Red light therapies have been used to treat several disease conditions in humans and in animal models for over 30 years including treatment of soft tissue injuries, diabetic wounds, radiation-induced ulcers, inflammatory and neurodegenerative conditions [6-11].

In the last 10 years, there has been an increasing interest in the use of 670 nm red light to manage retinal injuries. Early reports demonstrated the ability of 670 nm red light to attenuate damaging effects of methanol intoxication in rat retinas and laser-induced injury in primate retinas [12,13]. More recent studies have shown that 670 nm light treatment provides significant protection to photoreceptors in retinas exposed to damaging levels of light [14-16]. We have previously reported that 670 nm light treatment is effective in maintaining retinal function, and in significantly reducing expression of markers of inflammation and oxidative stress in rat retinas damaged by bright white light exposure [15-17]. Although the underlying mechanism of its therapeutic effect remains controversial, current evidence suggests that 670 nm light has a direct effect on the key mitochondrial enzyme, the cytochrome *c* oxidase (CCO). Upon absorption of 670 nm wavelength light, the activity of CCO increased along with energy production in the form of ATP [18,19]. Although unproven, this process is also thought to stimulate signalling pathways resulting in improved mitochondrial energy metabolism, antioxidant production, cell survival and regulation of non-coding RNAs [17,20].

Prolonged exposure to an elevated concentration of oxygen (hyperoxia) is toxic to a number of tissues in the respiratory and central nervous systems [21,22]. In the retina, early investigations in neonatal and adult rabbits and in adult mice demonstrated that exposure to hyperoxia can cause severe damage to photoreceptors [23-25]. The most commonly-observed pathological effects in the hyperoxia-induced mouse model are photoreceptor and endothelial cell death, increased inflammation, oxidative stress, erosion of the blood-retina barrier (BRB) and loss of functional vision [26,27,29-31]. These changes are site-specific, such that the irreversible loss of photoreceptors is most prominent in the circumscribed area of the inferior retina, approximately 500 μm from the optic disk [32].

Although the precise mechanism for the photoreceptor vulnerability to hyperoxia is not completely understood, it is postulated to be initiated and sustained by a “toxic cycle” of events [26], involving hyperoxia-induced oxidative stress in the outer retina through generation of free radicals and reactive oxygen species (ROS). Given the ineffective or lack of autoregulatory mechanism for the oxygen supply in the outer retina [33], prolonged exposure to hyperoxia will inevitably cause an increase in ROS production by the photoreceptors. Generation of ROS will subsequently trigger various pathological changes, such as oxidative damage, particularly in photoreceptors. These light sensitive cells contain a large number of polyunsaturated fatty acid (PUFA), that are also highly sensitive to ROS [34]. The hyperoxia-induced depletion of photoreceptors can also contribute to the rise of oxygen tension, ROS accumulation which in turn leads to further cell death, and progression of degeneration. These changes are also featured in the later stages of Age-related Macular Degeneration (AMD) and Retinitis Pigmentosa (RP) [35,36], suggesting that hyperoxia may be a suitable model for investigating novel rescue strategies to slow down the progression of these diseases.

In this study we aimed to examine the effects of pretreatment with 670 nm light on levels of oxidative stress, apoptosis and inflammatory response in the hyperoxia-induced retinopathy in adult mouse retina.

## Results

### Photoreceptor survival

**Figure 1:**
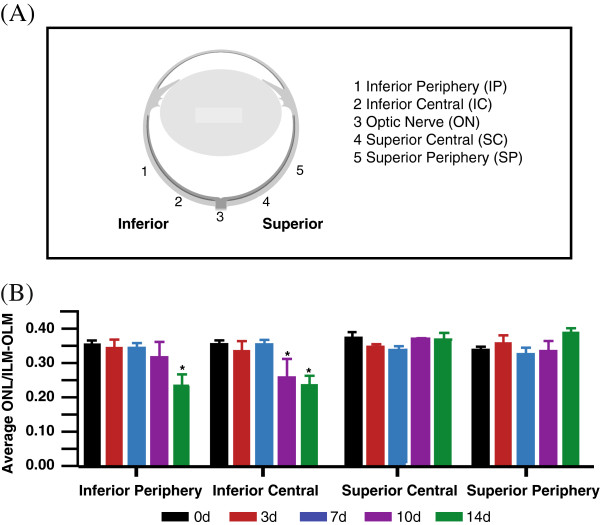
**Change in ONL thickness with exposure to hyperoxia.** Quantitative analysis of the impact of hyperoxia exposure (75% oxygen) to the photoreceptor population was performed in retinas of adult C57BL/6J mice (n = 12). **(A)** The average thickness of the outer nuclear layer (ONL) was sampled from four main areas; the inferior periphery, inferior central, superior central and superior periphery of the retinas from 0d control (black), 3d (red), 7d (blue), 10d (purple), and 14d (green). **(B)** Significant thinning of the ONL is evident by 10d of hyperoxic exposure, specifically in the inferior central area of the retina. At 14d in hyperoxia, depletion of the ONL has spread from the inferior central to the inferior periphery. The error bars representing the ± SEM. *Statistically significant thinning of the ONL (*p* < 0.05) compared to control animals.

Firstly, we evaluated the time course of the effects of hyperoxia on photoreceptor survival across the retina (Figure 1A). As depicted in Figure 1B, there was no significant change in outer nuclear layer (ONL) thickness in the superior retina at any of the timepoints. In the inferior retina, there was no significant change in ONL thickness following 3d and 7d exposure to hyperoxia. However, a significant reduction in ONL thickness was observed in the inferior central retina at 10d and 14d exposure (*p* < 0.001). In the inferior peripheral retina, a small (not significant) reduction in ONL thickness was detected at 10d followed by a significant thinning at this location at 14d exposure (*p* < 0.05).

**Figure 2:**
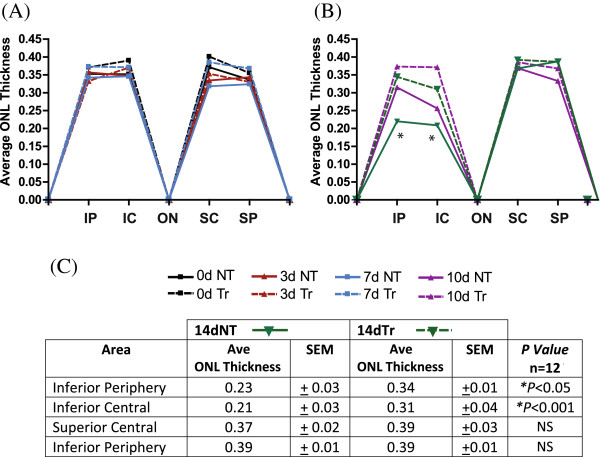
**Quantitative assessment of the effects of 670 nm light on the ONL thickness of hyperoxic mice.** The average thickness of the ONL was measured along the retinas of the nontreated (NT), hyperoxia-exposed (solid colored lines) and 670 nm-treated (Tr), hyperoxia-exposed mice (broken colored lines) from **(A)** 0d, 3d, 7d and **(B)** 10d and 14d groups. The retinas from 0d groups (NT, solid black line and Tr, broken black line) served as controls. **(C)** At 14d, the ONL of the Tr group (broken green line) in the inferior region was significantly thicker than the NT retinas (solid green line). *Statistically significant difference (*p* < 0.001) compared to the 670 nm light-treated retinas of the same time point.

**Figure 3:**
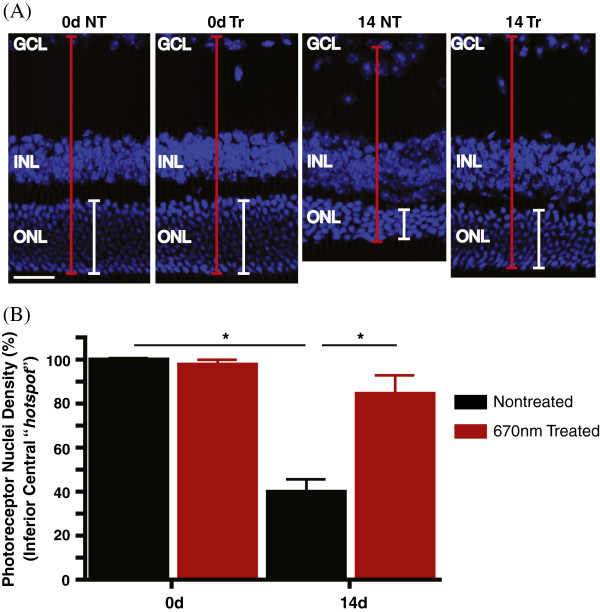
**Effects of 670 nm light treatment on the population of photoreceptors hyperoxic mice. (A)** Representative micrographs of retinal sections showing the nuclear layers stained with Bisbenzimide (blue). To account for obliquely cut retina and possible artefacts, the ratio of the ONL (white line) to the OLM-ILM (red line) was used for analyses. **(B)** Quantitative analysis of photoreceptor nuclei density in the inferior central region of NT group (black bar) and Tr mice (red bar). Compared with the nontreated retinas at 14d hyperoxia (14dNT), treatment with 670 nm red light induced a significant level of photoreceptor nuclei density preservation in the 14dTr group (*p < 0.001). Scale bar 50 μm in **A**.

Secondly, to further explore the regional effects of photoreceptor loss, we measured the ONL thickness and photoreceptor nuclei density at 1 mm intervals along the vertical meridian of the retina in both groups, nontreated (NT) and treated (Tr), at all timepoints. These data are summarized in Figures 2 and 3. The results showed no significant difference in retinal thickness and photoreceptor nuclei density in the NT and Tr groups at 0d (Figure 2A; solid black *vs* broken black lines and Figure 3A-B). Following 3d exposure to hyperoxia (Figure 2A; solid red *vs* broken red lines), there was also no change in the ONL thickness between NT and Tr groups. At 7d, treated animals (Figure 2A; solid blue line) showed a thicker ONL, but the difference between these and the 7dNT animals (Figure 2A; broken blue line) is not statistically significant (n = 12). After 10d exposure, the ONL is thinner in the inferior retina in both groups (10dNT and 10dTr) but indicates no significant difference between these groups (Figure 2A; solid purple *vs* broken purple lines). However, after 14d exposure to hyperoxia the ONL in the inferior retina is significantly thicker in 14dTr (Figure 2B-C, broken green line, Figure 3A) animals compared to 14dNT (Figure 2B-C, solid green line and Figure 3A) (*p* < 0.001). There is no significant difference in ONL thickness in superior region of the retina (central and periphery) in these groups (Figure 2B). Furthermore, the photoreceptor nuclei density counts in the inferior central area (Figure 3B) revealed a 60% decline in nontreated retinas (black bar) following 14d of hyperoxia exposure; while the 670 nm-treated groups (red bar) only showed a reduction of 15% compared with control baseline. The difference between 14dNT and 14dTr groups is statistically significant (*p* < 0.01).

### TUNEL (cell death) analysis

**Figure 4:**
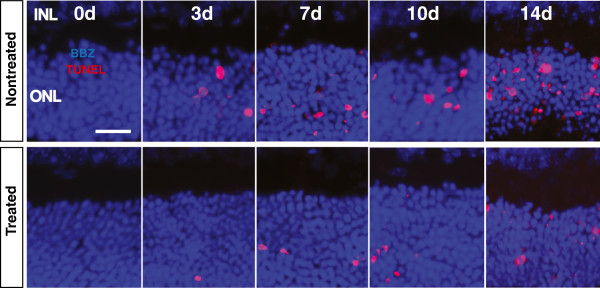
**Effects of 670 nm light pretreatment on photoreceptor cell death following hyperoxia.** Representative images of TUNEL-stained sections sampled from the inferior region of the NT and Tr retinas exposed to hyperoxia at different timepoints. TUNEL + labelling (red) was undetectable in the NT and Tr retinas from 0d (controls). A small population of TUNEL + photoreceptors was present in 3dNT retinas and continued to increase in number from 7, 10 and 14 days of exposure to hyperoxia. TUNEL + cells are also present in the 670 nm light-treated groups with significantly reduced number. The blue staining is Bisbenzimide, a DNA-specific stain, identifying the nuclear layers of the retina. Scale bar 25 μm.

**Figure 5:**
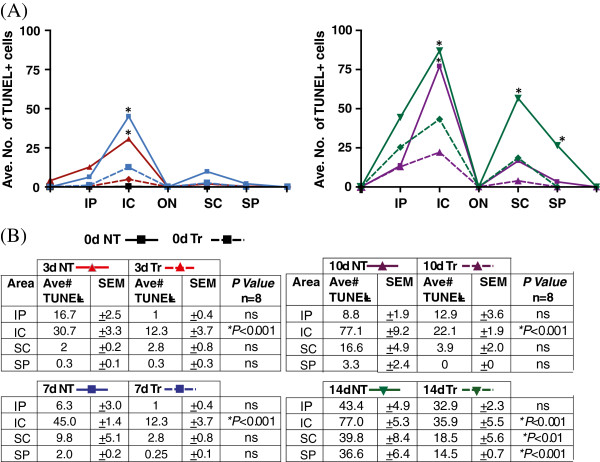
**Quantitative analyses of photoreceptors death. (A)** TUNEL + photoreceptor cells were counted across the retina (from the inferior to superior) in 0d, 3d, 7d, 10d and 14d animals and data from Nontreated (solid colored lines) and 670 nm Treated (broken colored lines) groups were compared. TUNEL + cells were present in the inferior central area (hotspot) of the nontreated retinas from 3d (solid red line) and the number of cell death increased as a function of hyperoxia-exposure time. **(B)** In the 14dNT retinas (solid green line), hyperoxia exposure caused widespread cell death that also affected the photoreceptors in the superior retina. By contrast, these numbers were significantly reduced by 670 nm light pretreatment. *Statistically significant difference (*p* < 0.001) compared to NT groups.

**Figure 6:**
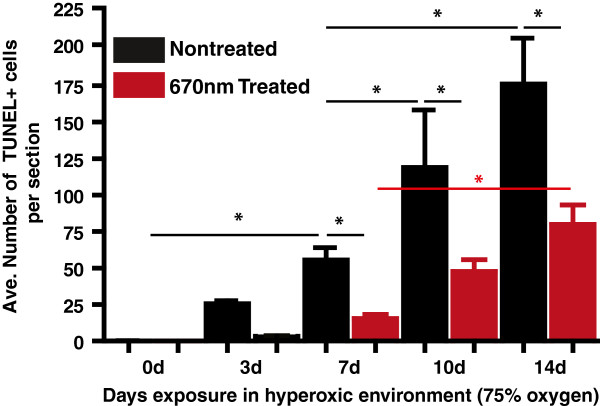
**Quantitative analysis of the average total number of TUNEL + cells.** Total counts of TUNEL + photoreceptors across the entire section of NT (black bar) and Tr (red bar) retinas from 0d, 3d, 7d, 10d and 14d groups. *Statistically significant difference (*p* < 0.05) between groups marked with black or red lines.

Findings from the terminal deoxynucleotidyl transferase dUTP nick end labeling (TUNEL) assay analysis are shown in Figures 4, 5, and 6. There was virtually no TUNEL + labelling detected in control retinas from 0dNT and 0dTr groups (Figure 4). In the 3dNT group there was a photoreceptor-cell specific death (Figure 4, red dots) confined almost exclusively to a circumscribed area approximately 500 μm inferior to the optic nerve head (the ‘hotspot’) (Figure 5A, solid red line) consistent with a previous report [30]; in 3dTr animals, only a small increase in TUNEL + cells was detected above baseline in the hotspot area (Figure 4, red dots and Figure 5A, red broken line). After 7d exposure to hyperoxia there is a further increase in incidence of TUNEL + cells in NT animals, in the hotspot area (45-fold above baseline) and in superior retina (Figure 5). In 7dTr animals, TUNEL + cells were detected in the hotspot (12.3-fold above baseline) and a few TUNEL + cells were also present in superior retina. Numbers of TUNEL + cells were further increased in both groups (NT and Tr) after 10d and 14d exposure to hyperoxia, in both the superior and inferior retina; however, the numbers of TUNEL + cells present in the 10Tr and 14dTr retinas were significantly lower compared with the 10dNT and 14dNT retinas, respectively (*p* < 0.001) (Figure 5, graph and tables).

The quantitative analyses for the average total number of TUNEL + photoreceptors in NT and Tr animals at different timepoints are summarized in Figure 6. The data show a sharp increase in TUNEL + cells associated with increased period of exposure to hyperoxia in NT retinas (3d to 14d, black bars). Although there is also an increase in TUNEL + cells in the Tr animals over the same period, TUNEL + cells only begin to accumulate in significant numbers until 7d exposure, and there are significantly fewer TUNEL + cells in Tr animals at each timepoint.

### Metabolic profile and oxidative stress

We used antibodies against Cox Va and acrolein to qualitatively and quantitatively evaluate the effect of 670 nm pretreatment on retinal metabolism and oxidative stress in animals exposed to hyperoxia. Because the damage resulting from hyperoxic exposure occurs mainly in a ‘hotspot’ in the inferior retina these analyses were carried out using images taken from that region. To further assess the level of oxidative stress, we used real time quantitative PCR to measure the heme oxygenase-1 (*Hmox-1*) mRNA expression.

### Retinal metabolic status (Cox Va)

**Figure 7:**
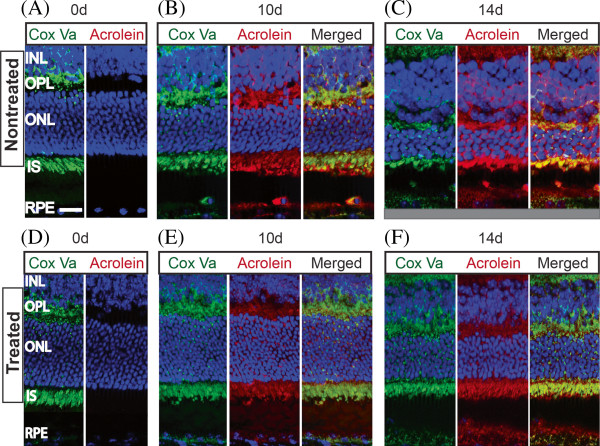
**Immunohistochemical analysis of Cox Va and acrolein. (A-F)** Effects of 670 nm light treatment on the metabolic status and oxidative stress profiles of the mouse retinas following exposure to 10d and 14d of hyperoxic exposure were examined by immunohistochemical staining. Cryosectioned retinas from the inferior region of the NT and Tr groups were labelled with marker for mitochondrial metabolism, Cox Va (green) and indicator of oxidative stress, acrolein (red). Compared to 0d control retina **(A)**, Cox Va labelling patterns (green) in the 10dNT and 14dNT were disrupted **(B**-**C**, green**)** but not in the 10dTr and 14dTr groups **(E**-**F**, green**)**. Exposure to hyperoxia caused a significant increase in the acrolein expression in 10dNT and 14dNT retinas **(B**-**C**, red**)** compared to 0d control **(D)**. The acrolein labelling was also present in the 10dTr and 14dTr groups but the intensity was much lower than the NT retinas **(E**-**F**, red**)**. Scale bar 25 μm.

**Figure 8:**
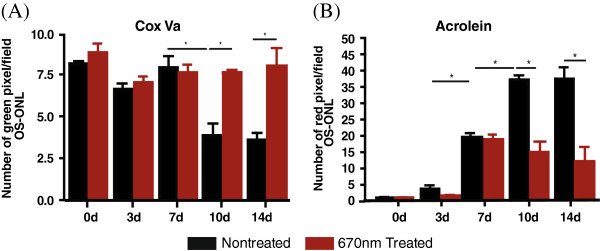
**670 nm light pretreatment regulates the expression of acrolein and Cox Va. (A-B)** Quantitative measurement of the fluorescent labelling intensity of retinal section from the inferior region labelled with mitochondrial Cox Va **(A)** and acrolein **(B)** from the nontreated (black) and 670 nm light-treated (red) hyperoxia-exposed retinas for 0, 3, 7, 10 and 14 days. *Statistically significant difference (*p* < 0.05) between groups highlighted by the solid black vertical line.

**Figure 9:**
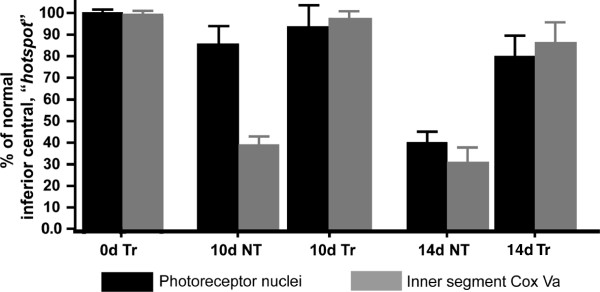
**Correlation between the population of photoreceptor nuclei (*black bar*) and the mitochondrial Cox Va immunostaining (*gray bar*) in the inner segments.** The level of Cox Va expressed by the mitochondria in the inner segments is proportional to the number of photoreceptor nuclei in all groups, except for retinas in 10dNT group. Animals that from 0dNT (nontreated, non-hyperoxic) group were used as baseline controls.

Qualitative analysis on immunostained retinal sections revealed an abundance of Cox Va with no discernible differences in the staining pattern between the 0dNT and 0dTr (Figure 7A and D, green). Antibody against Cox Va strongly labelled the mitochondria in the inner segments (IS) of the photoreceptors and was also detected in the outer plexiform layer (Figure 7A, green) as well as the inner retina (not shown). Quantitative measurement of Cox Va showed similar levels of immunoreactivity in the sampled areas at 0, 3 and 7 days of exposure to hyperoxia (Figure 8A) and no difference was detected between the Tr and NT groups. At 10d, the inner segments (IS) of the NT retinas were significantly disrupted and shortened, and reduced amounts of Cox Va immunoreactivity were detected (53% decline, *p < 0.05*) were detected compared to 10dTr group (Figure 7B, green; Figure 8A). In 10dTr retinas, the photoreceptor IS were less disrupted (Figure 7E, green) and showed levels of Cox Va immunoreactivity that were comparable with retinas at 7d (Figure 8A). After 14d exposure to hyperoxia, photoreceptor IS were shortened in NT animals, but retained good morphology in Tr retinas. However, levels of Cox Va immunoreactivity in the 14dTr animals were similar to those in the 10d groups (Figure 8A). Conversely, to determine the level of Cox Va expressed by surviving photoreceptors in the hotspot area following hyperoxic exposure of the nontreated and 670 nm-treated groups, Cox Va immunostaining in the IS was normalised to the number of photoreceptor nuclei. There was a correlation observed between the population of photoreceptor nuclei and the level of Cox Va expressed in the IS. The level of Cox Va labelling is proportional to the number of photoreceptors in early timepoints (results not shown) and in retinas with prolonged hyperoxic exposure, except for retinas from 10dNT group (Figure 9). In the 14dNT retinas, the reduction of photoreceptor nuclei density (Figure 9, black bar) correlated with the reduction of the level of Cox Va expression in the IS (Figure 9, gray bar). By contrast, the number of photoreceptor nuclei and the level of Cox Va immunoreactivity in all 670 nm-treated groups were close to control baseline levels (0dNT, non-hyperoxic, nontreated).

### Oxidative stress status (acrolein)

Baseline immunoreactivity to acrolein was determined from control retinas (0d; Figure 8B, red). A 3-fold increase in acrolein immunoreactivity above baseline was observed in 3dNT retinas (Figure 8B, black bar), increasing to 20-fold at 7d, 36-fold at 10dNT and 37-fold in 14dNT. In contrast, there was no significant increase of acrolein immunoreactivity in 3dTr retinas above baseline (Figure 8B, red); after 7 days of exposure to hyperoxia there was a 20-fold increase in acrolein immunoreactivity above baseline, comparable with 7dNT retinas. However, acrolein immunoreactivity was maintained at this level at the later timepoints in the Tr groups (10dTr and 14dTr).

### Gene expression

Expression of *Hmox-1*, *C3* and *Fgf-2* genes were analysed by quantitative RT-PCR to monitor the hyperoxia-induced stress responses of the NT and Tr retinas.

### Hmox-1

**Figure 10:**
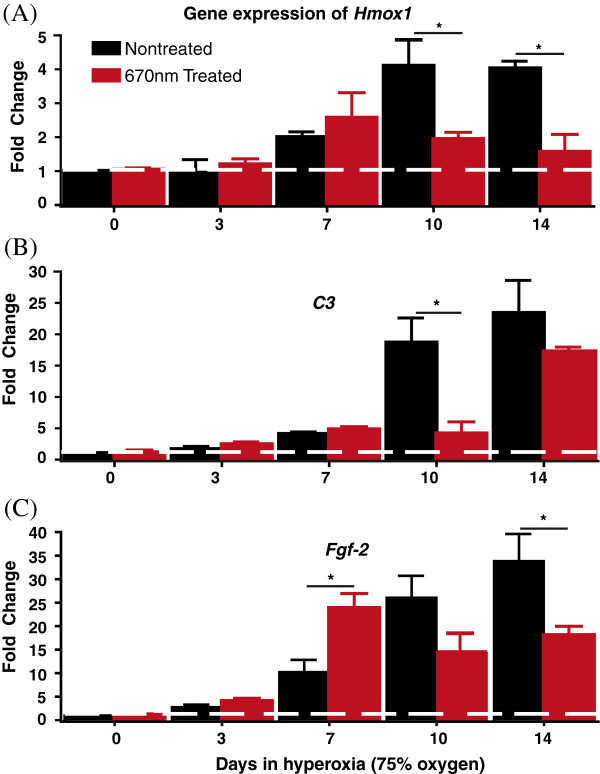
**Quantitative gene expression analyses. (A-C)** Differential expression profiles of oxidative stress-inducible **(A)***Hmox-1*, **(B)** proinflammatory *C3* and **(C)** neuroprotectant *Fgf-2* mRNA transcripts were measured using real time quantitative PCR. cDNAs from NT (black bar) and 670 nm-Tr (red) C57BL/6J retinas exposed to 0d, 3d, 7d, 10d 14d hyperoxia were used for quantitative analysis. The results were averaged from three independent experiments. The broken horizontal white line represents the fold change (FC) of 1 which means no change in the gene expression. The fold change of >1 represents gene upregulation while FC < 1 indicates downregulation. *Statistically significant difference (*p* < 0.05) between groups highlighted by the solid black horizontal line.

There was no significant change in *Hmox-1* gene expression after 3 days of exposure to hyperoxia, in either NT (black bar) or Tr (red bar) animals (Figure 10A). By 7 days exposure, there was a 2 to 2.75-fold up-regulation in *Hmox-1* expression in both 7dNT and 7dTr animals (difference not significant). By 10 days, there was a 4-fold up-regulation of *Hmox-1* expression in NT retinas, while in 10dTr retinas, levels of *Hmox*-1 expression remained at around 2-fold above baseline. Similar data were obtained from the 14d animals. The levels of *Hmox-1* expression in the Tr animals were significantly less than in the NT animals at both 10d and 14d (*p* < 0.05).

### C3

Expression of C3 increased over longer periods of hyperoxia exposure (Figure 10B) in both Tr and NT animals. In the NT group (black bars), this increase above baseline was significant at 7d and 14d. At 10d and 14d, there were significantly higher levels of *C3* expression in NT retinas (black bar) compared to Tr retinas (red bars) (*p* < 0.05).

### Fgf-2

A small and not statistically significant upregulation of *Fgf-2* was detected at 3d in both Tr (Figure 10C, red bar) and NT animals (Figure 9C, black bar). After 7d hyperoxia, there was a 10-fold up-regulation of *Fgf-2* in the NT retinas, compared with a 26-fold upregulation in Tr animals. Levels of expression were significantly different between 7dTr and 7dNT animals (*p* < 0.05). Levels of *Fgf-2* continued to increase in NT animals at 10d and 14d; however, in Tr animals there was a fall in *Fgf-2* expression at 10d to approximately 15-fold above baseline, which was maintained at 14d in the Tr group. At 10d and 14d, expression levels of *Fgf-2* in NT retinas were higher compared to Tr retinas, showing significant difference at 14d (*p* < 0.05).

## Discussion

These findings corroborate earlier studies showing that hyperoxia causes oxidative stress, photoreceptor-specific cell death, and retinal inflammation [30,32,38]. Those studies show that (i) hyperoxia-induced changes are evident by 3d exposure to hyperoxia, (ii) that there is a focal point of pathology in the inferior central retina, and (iii) this pathology spreads to other regions of the retina over time.

In addition, the present findings show that pretreatment with 670 nm light effectively offsets photoreceptor death induced by oxidative stress. The data demonstrate that pretreatment with 9 J/cm^2^ of 670 nm once daily for 5 consecutive days in this model: (i) delays the onset, and slows the progression of photoreceptor death, and (ii) downregulates the associated oxidative stress markers, acrolein and *Hmox-1*. This decrease in oxidative stress leads to the attenuation of damage to the retina and the reduction of expression of proinflammatory *C3* expression and reduced expression of the stress-inducible neuroprotectant, *Fgf-2.* Significantly, treatment of 670 nm light alone did not cause measurable damage in non-challenged retinas.

### 670 nm light treatment promotes photoreceptor survival

The timecourse of photoreceptor degeneration in this model has been reported previously [30]. In the present study, we find that the severity of the hyperoxia-induced damage in photoreceptors is significantly reduced, but not prevented, by pretreatment with 670 nm red light. We used two methods to quantify damage to photoreceptors: measures of ONL thickness, and counts of numbers of apoptotic (TUNEL positive) photoreceptors. Analyses of ONL thickness, the cumulative measure of cell loss, indicates that 14dTr animals have an ONL thickness profile approximating that of 10dNT animals (Figure 2), suggesting that in this model 9 J/cm^2^, 670 nm light treatment slows hyperoxia-induced degeneration by around 4 days. Counts of apoptotic photoreceptors (Figures 4 and 5) along the vertical meridian, a definitive indicator of the rate of photoreceptor loss, support this interpretation. The levels of cell death in 14dTr and 10dNT retinas; 10dTr and 7dNT were almost identical (Figures 4, 5 and 6), suggesting that 670 nm light delays progression of hyperoxia-induced cell death.

### 670 nm light attenuates oxidative damage

Hyperoxic exposure was previously demonstrated to induce oxidative stress, which differentially affects photoreceptors [31,38,39]. The high PUFA content, increased oxygen tension in the outer retina, and the continuous exposure to light render the photoreceptors vulnerable to oxidative stress [40]. The present data confirms those observations and indicates that irradiation with 670 nm light ameliorates oxidative stress such that there are minimal changes in cytochrome oxidase immunoreactivity, and lower levels of expression of the oxidative stress indicators, acrolein and *Hmox-1*.

Acrolein, the by-product of lipid peroxidation, and the stress-inducible *Hmox-1* gene are both reliable indicators for oxidative stress and have been linked to a range of degenerative conditions, including AMD [37,41]. In the present study, we observed changes in acrolein (Figure 8B) and *Hmox-1* (Figure 10A) expression profiles indicative of oxidative stress at 7 days of hyperoxia exposure (both NT and Tr). At 10 and 14 days, the NT retinas continued to show upregulation of these markers, implicating progression of oxidative stress. However, in the treated retinas in these time points (10dTr and 14dTr) there were no further increases in acrolein and *Hmox-1* expression levels. In addition, the intensity of immunolabelling for cytochrome oxidase in these retinas remained unchanged, suggesting that 670 nm light treatment maintains mitochondrial metabolism, leading to the attenuation of hyperoxia-induced oxidative damage.

Mitochondrial dysfunction increases oxidative stress, and has been implicated in the development of several retinal degenerative conditions including AMD, diabetic retinopathy and glaucoma [42-45]. Therefore, any treatment strategy aimed at preventing oxidative stress may prove beneficial in slowing the progression of retinal degeneration. Several reports showed that 670 nm light treatment reduces oxidative damage, including a rat *in vivo* model of methanol-induced retinal toxicity [12] and animals exposed to damaging bright light [16,17]. In the latter study, the levels of expression of the oxidative damage indicators, *Hmox-1*[17] and inducible nitric oxide synthase [16] were downregulated following 670 nm light treatment. Similarly, it has been reported that transcranial delivery of 670 nm light attenuates secondary oxidative damage in rat optic nerve following partial transection [46]. Moreover, findings from studies of Parkinson’s disease [5,47] and Multiple Sclerosis [9] also show that treatment with 670 nm light mitigates oxidative stress in these experimental models. Taken together, these observations provide strong evidence that 670 nm light ameliorates oxidative damage in disorders where oxidative stress plays a key role in the progression of pathology.

### 670 nm light mitigates C3 expression

The present study finds that exposure to hyperoxia causes a 20-fold upregulation of the complement component gene *C3* in NT retinas, by 10d exposure; this upregulation occurs in conjunction with high levels of expression of the oxidative stress markers, and increased rate of cell death (Figure 5). However, in the treated retinas there was a much smaller rise in C3 expression (5-fold in 10dTr retinas, Figure 10B). This finding is consistent with reports from studies carried out in aged rodents [48] and in rats exposed to damaging bright light [15,17].

Dysregulation of *C3* plays a highly significant role in retinal diseases, and dysregulation of complement is highly associated with risk of AMD [49,50,52]. Several lines of evidence suggest that oxidative stress triggers complement activation [53,54], which in turn mediates local inflammation. Tissue damage may result from cell-mediated attack, or by the formation of membrane attack complex, which kills cells. The low levels of *C3* expression in the treated retinas in this study, suggest that 670 nm light is a potential therapeutic tool applicable to retinal degenerative conditions where inflammation is involved, such as AMD.

### 670 nm light treatment reduces retinal damage

Several studies of retinal degeneration report an increase in expression of neuroprotective factors such as *Fgf-2* over time, which protects the retina by delaying, or in some cases, reducing photoreceptor cell death [51,55-57]. It has been reported previously that exposing the retina to a hyperoxic environment induces changes in the expression of *Fgf-2*[29,55]. The current study also demonstrates that *Fgf-2* expression is modulated by 670 nm light in hyperoxic conditions, although the effect is complex.

In this model, cell death in the ONL increases over time spent in hyperoxia, and at the 7d timepoint there are high levels of cell death in the NT group, and only low levels of cell death in the Tr group (Figures 5 and 6). Consistent with a role in neuroprotection, *Fgf-2* expression is higher in the 7dTr group (low levels of cell death) compared with the 7dNT group (Figure 10C). However, *Fgf-2* expression is significantly *lower* in 10dTr animals (compared with 10dNT) which is consistent with a finding that by 10d exposure to hyperoxia there are high levels of cell death, even in the retinas of treated animals. Beyond this timepoint *Fgf-2* expression remains approximately static. The explanation for the lowering of levels of *Fgf-2* in 10dTr and 14dTr is not immediately apparent, but may reflect the limitations of the efficacy of 670 nm light in offsetting the effects of hyperoxia. However, the reasons why 670 nm light treatment reaches a plateau, or declines, at this stage cannot be explained until we have a better understanding of its mechanism of action.

### 670 nm light in hyperoxia-induced retinal degeneration

The present findings indicate that the mechanism of action of 670 nm light is likely to involve signalling pathways activated by oxidative stress in the retina. It is generally accepted that oxidative stress initiates and facilitates the progression of hyperoxia-induced damage in the central nervous system and in the retina [31,58]. High oxygen tension is believed to induce metabolic disturbances in the PUFA-rich photoreceptor outer segments that trigger formation of ROS [35]. Accumulation of ROS leads to oxidative stress, and in turn to inflammation, and ultimately loss of functional vision [59,60]. Exposure of the retina to 670 nm light has been suggested to initiate key signalling pathways that activate transcription factors [61] to modulate pro/anti-oxidant balance in tissues [46], inflammatory responses [16,17] and cytoprotection [12-15]. The present results support those findings in that the data indicates that 670 nm light treatment limits oxidative stress and proinflammation, reducing retinal stress and cell death.

Several reports have suggested the important role of mitochondria in mediating the effects of 670 nm light irradiation [18,28]. First, the mitochondrial cytochrome oxidase has been proposed as the endogenous primary photoacceptor molecule in the reception of 670 nm light triggering secondary cellular processes such as enhanced energy-rich adenosine triphosphate (ATP) synthesis and other various modulatory effects [18,20]. Second, 670 nm light was suggested to influence retrograde signalling between the mitochondria and the nucleus, resulting in enhanced synthesis of DNA and RNA [28]. Third, the intra-mitochrondrial activities of nitric oxide (NO) and the cytochrome oxidase have recently been proposed as a novel mechanism of action of 670 nm light [62,63]. NO controls oxygen consumption of the tissue through reversible and competitive binding to cytochrome oxidase [64]. In a recent review, Poyton and Ball provided a mechanistic explanation suggesting that 670 nm light may cause dissociation of NO from the cytochrome oxidase complex allowing NO to bind effectively to oxygen during tissue damage [65].

## Conclusion

Here, we have demonstrated that hyperoxia-induced oxidative stress, cell death, retinal stress and inflammation were significantly mitigated, but not abolished, by 670 nm light treatment. Oxygen toxicity has been shown to cause oxidative stress and inflammation triggering photoreceptor cell death which are also implicated in the late stages of retinal degenerative conditions in humans, such as AMD, RP and Retinopathy of Prematurity (ROP) [66-68]. Hence, 670 nm red light treatment has the potential to slow the progression of retinopathies and conditions involving oxidative stress.

## Methods

### Animals

All procedures were in accordance with the Association for Research in Vision and Ophthalmology (ARVO) Statement for the Use of Animals in Ophthalmic and Vision Research, and with the requirements of The Australian National University Animal Experimentation Ethics Committee. C57BL/6J mice aged P90 (n = 12/group) of mixed sexes were reared in low (5 lx) light levels with a 12 h light, 12 h dark cycle. Food and water were available *ad libitum*.

### Oxygen damage (hyperoxia) and tissue collection

Animals were divided into 2 groups: 670 nm light-treated (Tr) and nontreated (NT). All experimental groups contained equal proportions of male and female C57BL/6J mice in all experiments. The treated animals received 670 nm light treatment prior to being placed in a clear plastic chamber, and exposed to 75% ± 0.5% oxygen, maintained using a computer-controlled feedback device (Oxycycler; Biospherix, Lacona, NY). The nontreated animals were placed in the oxygen chamber the same time as the 670 nm light-treated groups. Oxygen exposure commenced at 9:05 AM in all experimental groups. The animals were sacrificed and tissues were collected at 5 time points 0 day (0d, control), 3 days (3d), 7 days (7d), 10 days (10d) and 14 days (14d) exposure to 75% oxygen. Control animals were sacrificed at 0d (no O_2_) following 670 nm light treatment (Treated Controls), or without the 670 nm light treatment (Nontreated Controls). Tissues were collected at 9:00 AM of each time points.

### 670 nm pretreatment paradigm

All experimental groups (NT and Tr) were transferred into a clear plexiglass. The Tr groups were taken into the treatment room prior to 670 nm light treatment procedure. The 670 nm LED array (Quantum Device, WI, USA) was positioned so that the mouse eyes were approximately 2.5 cm away from the light source. Animals were exposed to 670 nm light for 3 minutes, once daily at 9:00 AM, for 5 consecutive days at 60 mW/cm^2^ delivering an energy fluence of 9 J/cm^2^ at eye level. Mice from the NT groups were transferred into separate room while the Tr animals were undergoing treatment.

### Experimental groups nomenclature

The nontreated animals are designated with the following group names: 0dNT, 3dNT, 7dNT, 10dNT and 14dNT. The 0dNT animals were not exposed to hyperoxia and did not receive 670 nm red light treatment. The 670 nm light-treated groups of each time point are designated as follows: 0dTr, 3dTr, 7dTr, 10dTr and 14dTr. The animals from 0dTr group were not exposed to hyperoxia but did receive 670 nm light-pretreatment.

### Tissue preparation

Mice were sacrificed by cervical dislocation. Excised retinas from the right eye of each animal were collected and stored in RNAlater® (Ambion, Applied Biosystems, Foster City, CA) overnight at 4°C for quantitative RT-PCR. Left eyes were marked at the superior aspect of the limbus for orientation, enucleated and immersion-fixed in 4% paraformaldehyde in 0.1 M phosphate-buffered saline (PBS) at pH 7.4 for 2 hours. Tissue was rinsed thrice in 0.1 M PBS and left in a 15% sucrose solution overnight for cryoprotection. The next day, eyes were embedded in Tissue-Tek OCT Compound (Sakura Finetek, Tokyo, Japan), and snap frozen in liquid nitrogen. The eyes were cryosectioned at 16 μm thickness, in the sagittal plane, to allow a dorsal to ventral observation of the retina using Leica CM1850 cryostat (Leica Microsystems, Nossloch, Germany). Sections were mounted on gelatin and poly-L-lysine-coated slides and dried overnight at 50°C before being stored at -20°C.

### Detection of cell death using TUNEL assay

Cryosectioned C57BL/6J mouse retinas from the NT and Tr groups at all timepoints were stained with TdT-mediated dUTP nick end labelling (TUNEL) using an established protocol [69]. To identify the layers of the retina, DNA-specific dye Bisbenzimide (BBZ) Hoechst (1:1000, Calbiochem, CA) was used. Quantitative assessment of cell death was conducted using parameters outlined in the following section.

### Retinal morphometric analyses

To quantify the level of hyperoxia-induced photoreceptor cell loss and apoptosis in the nontreated and treated groups, we used 2 methods: outer nuclear layer (ONL) thickness measurement and counts of photoreceptor nuclei density and TUNEL-positive (+) cells. Measurements of ONL thickness and counts of photoreceptor nuclei density were done in four main areas of the retina (Figure 1A); the inferior periphery (IP), the inferior central (IC), superior central (SC) and superior periphery (SP), using digital photomicrographs taken from the bisbenzimide-stained sections. In at least 4 retinal sections per animal, 10 measurements of ONL per section were obtained. To account for obliquely cut retina, we measured and recorded the thickness of the ONL, as well as the thickness of the retina, from the inner to the outer limiting membrane (OLM-ILM). The ratio of the ONL to the OLM-ILM was used for analysis. ImageJ software (National Institute of Health) was used to count photoreceptor nuclei density across the four main areas of the retina. The values obtained were normalised against the number of photoreceptor nuclei in the nontreated control retinas (0dNT) and the results were expressed in percentages. Secondly, the number of TUNEL + cells in the photoreceptor layer was counted across the retina, from inferior to superior, and either expressed per unit area and/or total number of TUNEL + cells, in 3 sections per animal. Counts of TUNEL + cells in control animals were used to determine baseline levels of cell death. Results from 12 animals from each group were averaged and analysed.

### Immunohistochemical analyses

Immunohistochemistry (IHC) was performed using standard protocols for fluorescent imaging. Briefly, frozen sections of the retina were placed in 70% ethanol followed by 2 rinses in 0.1 M of PBS. All sections were permeabilised with an antigen retrieval solution (Immunosolution Pty Ltd, Australia) for 45 mins at 37°C, followed by blocking with 10% normal goat serum (Sigma Aldrich, St Louis, MO, USA) for 1 h before incubation with the primary antibody for 24 h at 4°C. Primary antibodies used are mouse monoclonal antibody against cytochrome oxidase Va (1:100; Mitosciences, USA) and rabbit polyclonal antibody against acrolein (1:500; Cell Sciences, USA). Following rinses with 0.1 M PBS, sections were treated with a secondary antibody of either rabbit IgG conjugated with Alexa Fluor 594 or mouse IgG conjugated with Alexa Fluor 488 (1:1000, Molecular Probes, OR, USA) for 24 h at 4°C before incubation with the DNA-specific dye bisbenzimide Hoechst (1:10 000) for 2 min. Slides were coverslipped using a mixture of glycerol gelatin (Sigma, St Louis, MO, USA) and water.

### Confocal and light microscopy analyses

Sections were examined, scanned and analysed using Carl Zeiss LSM 5 Pascal confocal microscope (Germany), LM Zeiss Apotome (Germany) and Zeiss Axioplan. During image collection, the photomultiplier settings and exposure times for each fluorescent channel were kept constant to allow comparison of protein levels between sections.

### Quantitative measurement on signal intensity of immunofluorescent sections

Digital images of the acrolein and Cox Va-labelled sections obtained from confocal microscopy were processed and analysed using ImageJ software. Consistency in the analyses was ensured by using identical parameters with respect to section thickness (z-stack), areas sampled for analysis, antibody concentrations, duration of incubation times, and microscope configuration.

Five identical fields of interest were chosen in the inferior region per retina (n = 3). During the image quantification, the described areas were marked by a rectangle drawn from either the tip of the outer segments to outer plexiform layer or along the inner segments. Intensity of fluorescence from each rectangle (one area) was measured and the value was plotted as number of pixels/field (Cox Va, *green*; acrolein, *red*). The values obtained from each animal were averaged and presented in a histogram.

### RNA isolation and cDNA synthesis

Total cellular RNA was extracted from individual retinas and purified using the RNA extraction kit protocol (RNAqueous-Micro kit; Ambion). The purified RNA was quantified on a spectrophotometer (ND-1000; Nanodrop Technologies, Wilmington, DE). The integrity of the samples was assessed using a bioanalyser (2100 Bioanalyzer; Agilent Technologies, Santa Clara, CA). The cDNA was synthesized by reverse transcription using reverse transcriptase or first strand cDNA synthesis kit following the manufacturer’s protocols (Superscript III; Invitrogen, Carlsbad, CA).

### Real time quantitative polymerase chain reaction (RT-qPCR)

Quantitative analysis of expression levels for genes *Hmox-1* (*Mm00516005*), *C3* (*Mm00437838*), *Fgf-2* (*Mm00433287*) and was determined by RT-qPCR using Taqman® probes that were combined with the Gene Expression Master-Mix (Applied Biosystems, Foster City, CA). StepOne Plus qPCR machine was used with the StepOne software v2.1 (Applied Biosystems) for analysis. Glyceraldehyde 3-phosphate dehydrogenase (*Gapdh*) was used as a reference gene. The variabilities were accounted for by performing the Taqman amplification assay in duplicates (individual sample variability) with triplicate biological samples to account for individual animal differences. The fold changes were determined using comparative cycle threshold (Ct; delta-delta ct).

### Statistical analyses

Data were analysed using non-parametric tests, Kruskal-Wallis followed by Mann–Whitney *U* or parametric ANOVA test with Bonferroni corrections. Results are presented as mean ± Standard Error of the Mean (SEM). *P* value of <0.05 was considered significant.

## Abbreviations

AMD: Age-related macular degeneration; ARVO: Association for research in vision and ophthalmology; ATP: Adenosine triphosphate; BBZ: Bisbenzimide; BRB: Blood-retinal barrier; CCO: Cytochrome *c* oxidase; Cox Va: Cytochrome oxidase Va; Ct: Cycle threshold; C3: Complement component 3; FC: Fold change; Fgf-2: Fibroblast growth factor 2; FR: Far red; Gapdh: Glyceraldehyde 3-phosphate dehydrogenase; Hmox-1: Heme oxygenase – 1; IHC: Immunohistochemistry; IC: Inferior central; IP: Inferior periphery; ILM: Inner limiting membrane; IS: Inner segments; mRNA: Messenger RNA; NIR: Near infrared; nm: Nanometer; NO: Nitric oxide; NT: Nontreated; OLM: Outer limiting membrane; ON: Optic nerve; ONL: Outer nuclear layer; OS: Outer segments; PUFA: Polyunsaturated fatty acid; qPCR: Quantitative polymerase chain reaction; ROP: Retinopathy of prematurity; ROS: Reactive oxygen species; RP: Retinitis pigmentosa; SC: Superior central; SP: Superior periphery; TUNEL: Terminal deoxynucleotidyl transferase dUTP nick end labeling; Tr: Treated.

## Competing interests

The authors declare that they have no competing interests.

## Authors’ contributions

RA participated and contributed substantially in the conception and design of the study; carried out all the experiments; analysed and interpreted the data and drafted the manuscript. RN made a substantial contribution to the design, analyses and interpretation of the data and involved in revising the manuscript. MR contributed to the analysis and interpretation of the data and involved in revising the manuscript. KV made a substantial contribution to the design, analysis and interpretation of the data and involved in revising the manuscript. JP made a substantial contribution to the design, analysis and interpretation of the data; involved in drafting and revising the manuscript for important intellectual content and has given the final approval of the version for submission. All authors have read and approved the final manuscript.
